# Brief mindfulness training enhances cognitive control in socioemotional contexts: Behavioral and neural evidence

**DOI:** 10.1371/journal.pone.0219862

**Published:** 2019-07-19

**Authors:** Jordan T. Quaglia, Fadel Zeidan, Peter G. Grossenbacher, Sara P. Freeman, Sarah E. Braun, Alexandra Martelli, Robert J. Goodman, Kirk Warren Brown

**Affiliations:** 1 Department of Psychology, Virginia Commonwealth University, Richmond, VA, United States of America; 2 Department of Contemplative Psychology, Naropa University, Boulder, CO, United States of America; 3 Department of Anesthesiology, Center for Mindfulness, University of California San Diego, CA, United States of America; 4 Department of Psychological Sciences, Northern Arizona University, Flagstaff, AZ, United States of America; Boston University, UNITED STATES

## Abstract

In social contexts, the dynamic nature of others’ emotions places unique demands on attention and emotion regulation. Mindfulness, characterized by heightened and receptive moment-to-moment attending, may be well-suited to meet these demands. In particular, mindfulness may support more effective cognitive control in social situations via efficient deployment of top-down attention. To test this, a randomized controlled study examined effects of mindfulness training (MT) on behavioral and neural (event-related potentials [ERPs]) responses during an emotional go/no-go task that tested cognitive control in the context of emotional facial expressions that tend to elicit approach or avoidance behavior. Participants (*N* = 66) were randomly assigned to four brief (20 min) MT sessions or to structurally equivalent book learning control sessions. Relative to the control group, MT led to improved discrimination of facial expressions, as indexed by d-prime, as well as more efficient cognitive control, as indexed by response time and accuracy, and particularly for those evidencing poorer discrimination and cognitive control at baseline. MT also produced better conflict monitoring of behavioral goal-prepotent response tendencies, as indexed by larger No-Go N200 ERP amplitudes, and particularly so for those with smaller No-Go amplitude at baseline. Overall, findings are consistent with MT’s potential to enhance deployment of early top-down attention to better meet the unique cognitive and emotional demands of socioemotional contexts, particularly for those with greater opportunity for change. Findings also suggest that early top-down attention deployment could be a cognitive mechanism correspondent to the present-oriented attention commonly used to explain regulatory benefits of mindfulness more broadly.

## Introduction

The ability to actively maintain mental representations of personal or social goals and the means to achieve them is a hallmark of mature behavior regulation (e.g., [1,2]) and key to success in a variety of life domains (e.g., [3,4]). In day-to-day life this cognitive control is supported by a collection of interacting control processes such as the maintenance of behavioral goals, selection of relevant information, and conflict monitoring and resolution [5]. Each of these control processes frequently interact with emotion [6,7], which can help or hinder successful cognitive control. For example, interest or curiosity can facilitate goal pursuit, and fear or anger can hinder it. Deficits in cognitive control are central to a variety of forms of maladaptive behavior, including risk-taking, addictions, and aggressiveness [8], and not coincidentally, they frequently occur when cognitive control is compromised by the need to process challenging emotional information [9].

Social situations involve unique challenges for regulating goal-driven behavior in the face of one's own and others' changing emotions. These dynamic situations often require the maintenance of cognitive control in the context of potentially interfering emotional information [10], such as others’ facial expressions; in the pursuit of social goals, such as a successful business negotiation or the peaceful resolution of a disagreement, others’ expressions of emotion may conflict with prepotent (habitual) tendencies to approach or withdraw from emotional cues in the situation. Consider the example of romantic partners in the midst of a relationship conflict. Each member of the couple typically seeks to achieve personal and/or relational goals by the end of the discussion, and to do so must carefully regulate their behavior when their partner expresses emotions that interfere with one's communication. Perhaps even more challenging, each must stay engaged when emotions are expressed that humans are biologically prepared to withdraw from (e.g., fear, anger). Most everyone has experienced such challenges and, at least on occasion, their cognitive control capacities have failed them, leading many to wish these capacities were stronger.

How can cognitive control in socioemotional contexts be strengthened? It has been argued that enhancing volitional attention to affective cues can facilitate cognitive control. In particular, training in *mindfulness*, classically described as a sustained, receptive, moment-to-moment attention to salient information (e.g., [11]), is thought to strengthen top-down attention—that is, selective allocation of attention driven by cognitive factors such as knowledge and goals [12]. Strengthening top-down attention in this way could enhance cognitive control by aiding the selection and maintenance of task-relevant information [13,14]. Whether mindfulness training has such an effect when facing the unique demands of socioemotional situations is unknown, however. The present research sought to address this question by examining effects of mindfulness training on neural and behavioral markers of cognitive control in a laboratory-based socioemotional context. Addressing this question will inform efforts to improve cognitive control in ways that commonly impact interpersonal well-being and success.

### Cognitive control in social situations

Success in many social interactions, whether professional or personal, often depends on the initiation and maintenance of mental representations of one’s implicit or explicit goals for those interactions. Such cognitive control may be particularly important for goal-driven responding in social contexts due to numerous emotional signals, such as others’ facial expressions, that can elicit automatic emotional reactions [15]. Indeed, the presence of others’ emotions in social situations can place unique demands on cognitive control. People tend to approach pleasant facial expressions (e.g., happy faces) and avoid unpleasant facial expressions (e.g., fearful faces; [9,16]), but such prepotent response tendencies may guide or conflict with goal pursuit, as the example of a romantic couple interaction above illustrates.

Specifically, we suggest that the presence of socioemotional information presents three interrelated challenges to cognitive control. First, facial expressions of emotion can change (e.g., appear and disappear) rapidly, yet these dynamics can provide important information relevant to social goal pursuit [17]. Thus, facial expression recognition and discrimination becomes a fundamental skill to support cognitive control of emotional and behavioral responses to facial expressions [10]. Second, cognitive control and emotion regulation frequently interact in socioemotional contexts, as emotional information can support or interfere with cognitive control. *Implicit-controlled emotion regulation* [18] is a form of rapid emotion regulation, often preceding explicit strategy use, that requires holding in mind rules for selecting or inhibiting behavior when encountering emotional information (e.g., others’ emotions) while monitoring behavioral performance in relation to those rules or goals. A third challenge to cognitive control in social contexts is monitoring of the conflict between one’s goal states and prepotent responses. This involves detecting conflict between habitual response tendencies and infrequent or new situational demands. It also includes the application of cognitive control to override automatic tendencies to others’ emotions through, for example, approaching unpleasant (e.g., fearful) facial expressions when called for.

### Mindfulness training as cognitive control training

We propose that mindfulness may be well-suited to meet these cognitive control challenges, primarily via an up-regulation of early top-down, goal-driven attention. Broadly speaking, mindfulness is theorized to promote cognitive control by refining attention to sensory (including emotional) cues that are implicated in the initiation and maintenance of executive attention [14]. In interpersonal contexts, basic recognition and discrimination processes depend on early top-down attention [19], so facial expressions of emotion may be more easily and accurately discerned when attention is heightened [20]. In this way, mindfulness may help to address the first challenge to cognitive control in social contexts noted earlier.

Addressing the second challenge, mindfulness may also support implicit-controlled emotion regulation by facilitating the processing of emotional information while holding rules or goals in mind. Specifically, the receptivity or openness to sensory cues that characterizes mindfulness may promote accurate and timely responses to emotional signals compared to processing that involves mentally elaborating on those cues or suppressing them [14]. As a form of attention deployment, mindfulness requires fewer cognitive resources than regulatory strategies such as reappraisal and suppression, which require more effortful cognitive engagement [21]. Indirect evidence in support of such efficient processing comes from attentional blink research showing that mindfulness training led to heightened perception of rapidly presented visual (letter) stimuli [5]. This finding is instructive, but to date there is no direct evidence that mindfulness training can enhance the processing of emotional information in ways instrumental to implicit-controlled emotion regulation and cognitive control in a socioemotional context.

The third challenge to cognitive control in socioemotional situations noted earlier concerns monitoring of the conflict between behavioral goals and inherent approach and avoidance tendencies. If, as we suggest, mindfulness conserves cognitive resources in the regulation of responses to others’ emotions, it may be well-suited for promoting conflict monitoring. Again, evidence from cognitive performance research supports this view. For example, Jha et al. [22] found that experienced meditators exhibited strong conflict monitoring performance on the Attention Network Task [23]. Tang et al. [24] further showed that brief (5-day) meditation training improved conflict monitoring on this task, while Teper and Inzlicht [25] found meditators to have better performance on the Stroop task perhaps because meditation training enhanced attention focus and reduced emotion reactivity or, as their evidence suggested, because they paid more attention to the emotions associated with making errors. Mindfulness training may similarly promote early regulation of habit-driven responses as well as between automatic approach- and avoidance-related tendencies in social contexts dense with emotional signals. Ultimately, such conflict monitoring could promote more context-sensitive responding to emotional signals in socioemotional contexts.

Mindfulness training effects have not been studied in socioemotional contexts, but evidence from a study of trait mindfulness is suggestive. Quaglia et al. [20] found that a basic operationalization of trait mindfulness predicted behavioral and neural indices of more time-efficient and accurate cognitive control performance while viewing human facial expressions of emotion in an emotional go/no-go task. Convergently, more mindful individuals showed amplified N200 and No-Go P300 event-related potentials (ERPs), which are neurophysiological signals occurring approximately 200 ms and 300 ms post-stimulus, respectively, that reflect better conflict monitoring in this socioemotional task environment.

The benefits of mindfulness training for cognitive control may be a direct result of how mindfulness is commonly trained. The “focused attention” form of mindfulness training entails sustaining voluntary attention to an intended object–often kinesthetic and proprioceptive sensations–while monitoring for distractions that draw attention away from the object and redirecting attention back to the intended object when indicated [26]. In this way, practice in focused attention trains cognitive control by requiring practitioners to monitor conscious experience as it is unfolding, and to refocus attention on the chosen object when they detect that their minds have wandered (e.g., [14]). Coupled with theory and evidence suggesting that mindfulness training strengthens prefrontal control mechanisms supportive of emotion regulation [13], prior research offers initial support to the hypothesis that focused attention-based mindfulness training will improve cognitive control in ways important for emotion regulation in socioemotional contexts.

### The present research

Careful, goal-driven attention appears key to regulating behavioral responses effectively in socioemotional situations. It is therefore important to ask what impact attention training has on cognitive control in response to socioemotional stimuli. The present study examined whether brief training (four 20-minute sessions) in a focused attention form of mindfulness would result in enhanced cognitive control of behavioral responses to facial expressions of emotion, as indexed by changes in behavioral and neural markers collected during performance of an emotional go/no-go task. Individuals were randomized to a brief mindfulness training (MT) condition or a structurally equivalent book listening control (BLC) condition, the latter to account for nonspecific effects related to expectations (e.g., reduction in stress), intervention setting, instructor contact time, demand characteristics, and learning processes (cf. [27–30]). At pre- and post-intervention, behavioral and neural responses were assessed during an emotional go/no-go task.

The emotional go/no-go task provides an environment wherein the three challenges to cognitive control in socioemotional contexts can be examined in a controlled manner–namely (a) effective identification and discrimination of facial expressions (in this study, happy, neutral, or fearful expressions); (b) adherence to rules for selecting or inhibiting behavior in the face of others’ emotions; and (c) successful monitoring of goal–prepotent tendency conflicts, whether from task demands or the presence of emotional information. In the task, participants are asked to respond quickly and accurately with a button press to target facial expressions, which constitute the majority of trials in the task, whilst withholding responses to nontarget facial expressions. Importantly, the emotional go/no-go task may not directly assess emotion regulation, given that others’ emotional expressions may not elicit emotions in perceivers. Instead, this task permits assessment of three challenges to cognitive control that may be instrumental to effective emotion regulation in socioemotional situations.

To examine whether MT, relative to BLC, could meet the first challenge concerning discrimination of facial expressions, we examined condition differences in d-prime, a commonly used index of accurate stimulus discrimination that accounts for response bias [10]. To assess whether MT, relative to BLC, could meet the second challenge of cognitive control in emotion-laden contexts, namely the processing of emotional information while holding rules or goals in mind (processes important for implicit-controlled emotion regulation), analyses of condition differences in the response time (RT) of correct responses (Go trials) and errors of commission on No-Go trials (false alarms; FAs) were performed. Both RT and FA rate are useful for indexing cognitive control during the emotional go/no-go task, since rapid and accurate performance concerns the maintenance of cognitive control in the presence of emotional information. An additional measure used was an efficiency score that combined RT and FA rate to account for speed/accuracy tradeoffs, namely the inverse efficiency score (IES; [31]). Whereas RT or FA rate alone do not account for performance strategies that favor speed or accuracy, the IES accounts for speed/accuracy tradeoffs to index efficient cognitive performance expressed in a single index [31].

Finally, to examine whether MT helped to meet the third challenge of interest here–namely concerning the success of monitoring and resolution of goal–prepotent tendency conflicts, both behavioral and neural indices were examined. In the emotional go/no-go, conflict monitoring includes approaching what is aversive, indexed by quicker RT to fearful faces in particular–as well as refraining from approaching what is pleasant, indexed by lower FA to happy faces. More specifically, faster RT to target fearful faces should be slower due to the need to override the prepotent tendency to avoid unpleasant stimuli, whereas FA rate should be higher to nontarget happy faces because of greater difficulty inhibiting approach responses to pleasant stimuli [16]. Neurophysiological response can provide a clear window into conflict monitoring and resolution processes. Such processes unfold very quickly, and ERPs derived from electroencephalographic (EEG) signals provide temporally precise measurement of cortical brain activity and have been shown valuable for identifying neural signatures of conflict monitoring in tasks such as the emotional go/no-go. Consistent with prior research examining ERPs in the emotional go/no-go task (e.g., [9]), as well as with our emotional go/no-go research on dispositional mindfulness [20], the N200 and P300 ERP components were selected to assess effects of MT (vs. BLC) on conflict monitoring and resolution.

The N200 is a negative-going ERP deflection found primarily over the fronto-central region of the scalp, theorized to index attentional monitoring of the discrepancy between internally-generated intended behavior and external task demands–that is, conflict monitoring [32,33]. As per its functional association with executive attention, the N200 has been localized to brain regions of the executive attention network [34]. Supporting the functional significance of the No-Go N200 in particular as a marker of conflict monitoring, peak amplitudes for the N200 have been found to be larger for trials involving conflict between prepotent and controlled behaviors [32]. In the emotional go/no-go task, these occasions occur when inhibition of responses is required (No-Go trials).

The P300 is also commonly measured over the fronto-central region, and the No-Go P300 in particular has been associated with the cancellation of intended, task-inappropriate behavior (button pressing) on No-Go trials [9,32,35]. The No-Go P300 may thus be an important complement to the N200 for examining executive resolution of conflict through inhibitory control. Yet because the N200 and P300 components reflect dissociable executive processes, MT-related changes in the efficiency of conflict monitoring as reflected in the N200 may not correspond with changes in the P300. Indeed, to the extent that MT-related changes in conflict monitoring (No-Go N200) reflect more resolution of conflict, this could lessen cognitive demand for subsequent behavioral inhibition. This reasoning was also given by Megías et al. [9], who found that individual differences in emotional intelligence were related to larger N200 amplitudes but not No-Go P300 amplitudes. Thus, we predicted that MT would be more likely to impact No-Go N200 than No-Go P300, although we examined training-related associations with the latter in an exploratory fashion.

Broadly speaking, and consistent with our earlier correlational research [20,36], we expected that MT would lead to enhanced cognitive control helpful for responding to socioemotional stimuli, here operationalized by pleasant (happy), aversive (fearful), and neutral facial expressions. This examination of behavioral and neural responses during the emotional go/no-go allowed us to address how MT meets the three challenges noted earlier. First, we expected that MT participants (vs. BLC participants) would demonstrate greater pre-post training improvements in discrimination of all three types of facial expressions in the emotional go/no-go task, as indexed by d-prime. Regarding the second challenge, we predicted that MT (vs. BLC) would produce more accurate and efficient performance across the various combinations of happy, neutral, and fearful targets/nontargets, as indexed by RT, FA rate, and their combination to account for speed/accuracy tradeoffs. These condition differences would speak most directly to the maintenance of goal-driven responding in the face of others’ emotions, reflecting cognitive changes likely supportive of implicit-controlled emotion regulation. Finally, addressing the third challenge, we expected that relative to BLC, MT would result in greater pre-post training increases in conflict monitoring, as indexed by behavioral and ERP indexes–specifically reflected in quicker RT to fearful faces and lower FA to happy faces, as well as amplified N200 generally or No-Go N200 specifically. Although the No-Go N200 is more commonly associated with conflict monitoring, our prediction included the potential for higher N200 generally since it also appears to reflect conflict monitoring [9,20]. We also explored whether MT would predict increased No-Go P300 amplitude, reflecting a later-occurring response inhibition on No-Go trials.

## Methods

### Participants

This research was approved by Virginia Commonwealth University's Institutional Review Board. Written consent was obtained from all study participants. A power analysis [37] determined that a sample size of 66 participants would be needed, using an average effect size from similar MT research [28,38], with statistical power of 0.80 and α = .05. A community sample of participants from the southeastern United States was recruited with advertisements for a free mindfulness meditation training course, plus monetary incentive ($100). Each participant was screened for age (18 to 60 years), lack of previous direct experience with meditation, and native English language-speaking. Only individuals in committed, romantic relationships (> 12 months, cohabitating) were recruited for other study purposes, though just one member of the couple completed the measures and procedures reported here. Additionally, participants were screened based on self-reported history of common neurological conditions (open-ended response), as well as yes/no questions regarding prior or current neurological, psychiatric, psychotropic medication use, substance abuse, or significant medical condition; a body mass index (BMI) < 32 to account for potential cognitive deficits of obesity [39]; and were primarily right-handed [40]to account for potential differences in electrocortical outcomes between left- and right-handed individuals [41]. After baseline, simple randomization via randomizer software was used to allocate qualifying participants to conditions.

**Fig 1:**
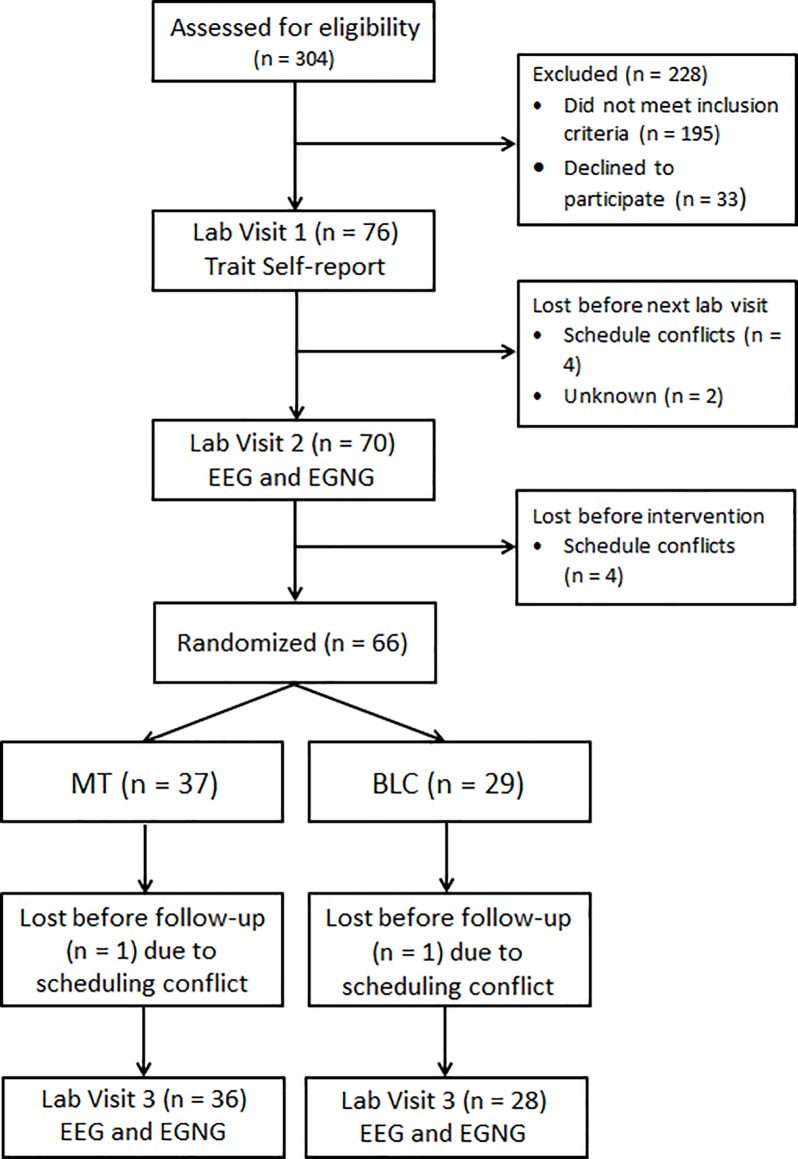
Flow of participants through the study. Sixty-four participants completed the intervention and laboratory assessments, but the total number of participants included in behavioral (or EEG) analyses was *n* = 34 (33) in MT and *n* = 26 (25) in BLC, based on those with valid data at both time points. EGNG = Emotional go/no-go.

Fig 1 presents the participant flow through the study. Using random assignment at the level of week of the study, more participants were by chance assigned to MT than to BLC, with 37 participants randomized to MT and 29 to BLC. In each condition, one participant was unable to complete the study because of scheduling conflicts. Two additional participants per condition did not have valid behavioral data at both time points because of procedural errors, and one additional participant per condition did not have valid EEG data due to equipment failures, leaving 34 (33 for ERP) participants for MT and 26 (25 for ERP) for BLC. Of participants reported on, 31% were male and 69% were female. Ages ranged from 20–59 years old (*M =* 30.58, *SD =* 9.10), with more than 75% of participants between the ages of 20 and 35. The race/ethnicity composition was as follows: 1.5% Asian or Pacific Islander, 3.2% Asian Indian, 6.3% Black/African American (non-Hispanic), 80.9% Caucasian/White, 1.5% Latino/Hispanic, 1.5% Native American, and 3.2% bi/multiracial.

### Intervention

Intervention guidelines for brief MT followed previous research by Zeidan and colleagues [28,29,38]. This brief MT intervention involved four 20-minute sessions of focused attention mindfulness meditation facilitated by a male instructor with over 8 years of mindfulness meditation experience, with progressively more self-directed practice from session to session. Participants randomly assigned to the BLC condition underwent a structurally equivalent procedure: four 20-minute sessions of listening to a book [42] read aloud by a male instructor on a neutral topic. Both procedures were delivered in groups of 2–6 participants at the same location at a local community center, occurred on the same days of the week, and introductory remarks provided participants the same benefit expectancy; all were advised that their particular training could reduce stress and promote well-being. No adverse events were reported in either condition, and all participants in the BLC condition were invited to participate in MT after study debriefing, though no further data were collected.

### Materials

#### Self-report measures

**Credibility and expected benefits.** To account for any differences in training credibility and expected benefits between intervention conditions, participants completed the Credibility/Expectancy Questionnaire (CEQ; [43]) immediately post-randomization and after receiving a description of their intervention. Sample Cronbach’s alphas were .90 (credibility) and .92 (expected benefits).

**Dispositional mindfulness.** The 14-item Freiburg Mindfulness Inventory (FMI; [44]) was used to assess the effect of MT on self-reported dispositional mindfulness (sample item: “When I notice an absence of mind, I simply return to the experience of here and now”). This measure was used to check for dispositional mindfulness differences in groups at baseline. Cronbach’s alpha for the FMI was .86 in this sample.

**State mindfulness.** The Attention subscale of the Practice Quality-Mindfulness questionnaire (PQ-M; [45]) served as a manipulation check of present-moment mindful attention during the final intervention session, directly preceding the post-training emotional go/no-go. Participants estimate the proportion of time for a given period that their experience reflected the statement, with choices from 0–100%. A sample item is, “During the activity, I attempted to return to my present-moment experience, whether unpleasant, pleasant, or neutral.” Sample Cronbach’s alpha was .89.

#### Emotional go/no-go task measures

**Fig 2:**
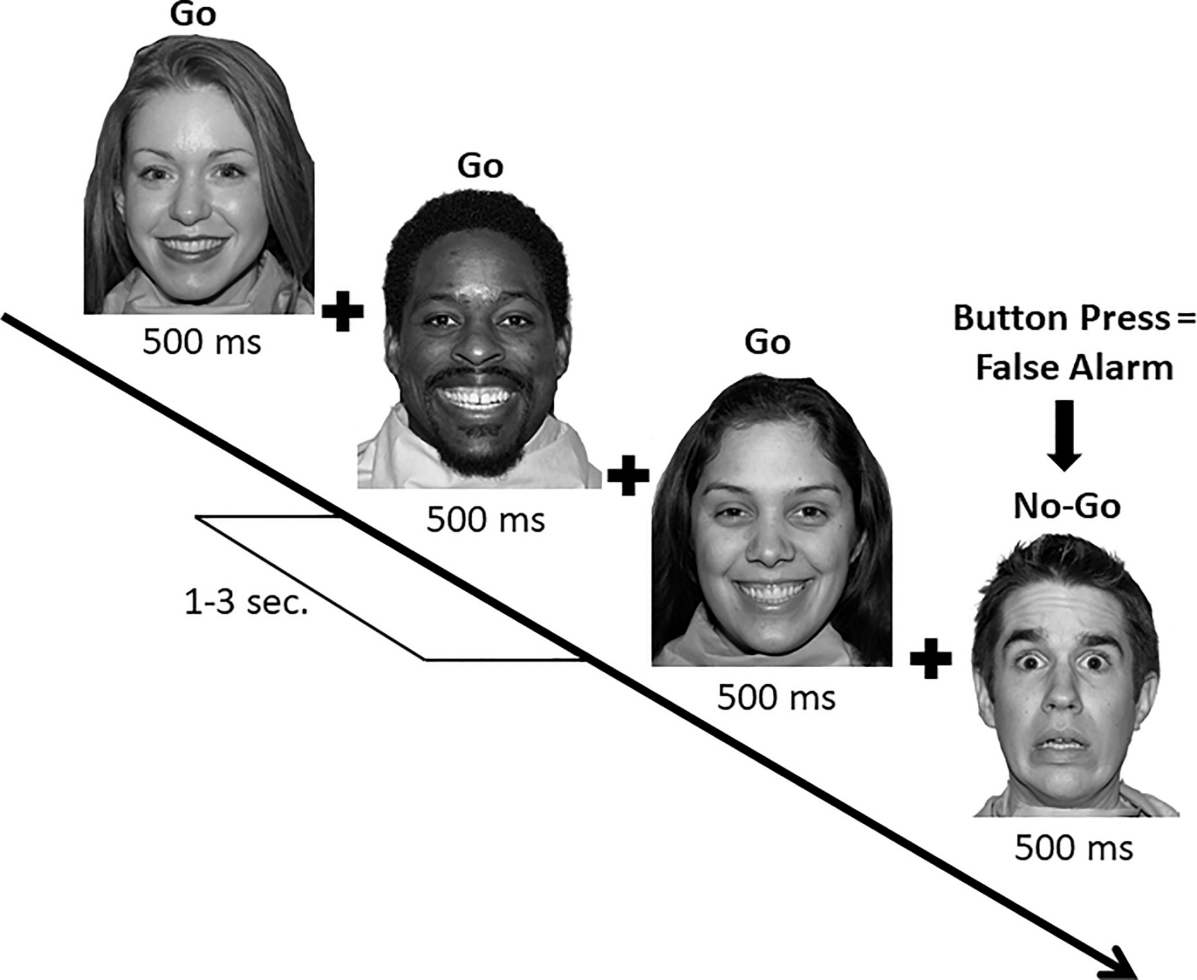
Temporal sequence of the emotional go/no-go task. In the block variation illustrated here, targets (go) are happy facial expressions and nontargets (no-go) are fearful facial expressions.

**Behavioral measures**. Socioemotional stimuli for the emotional go/no-go task were selected from the NimStim Face Stimulus Set [46] as previously used in emotional go/no-go studies [16,20,36]. Twelve models from the NimStim Set (6, 8, 11, 14, 15, 16, 25, 27, 36, 39, 43, and 45) included African American, Asian, and Caucasian males and females expressing happy, neutral, or fearful facial expressions. Prior to use, images were grayscaled and normalized for luminance. Following Hare et al.’s [16] procedure, with minor technical adjustments for an ERP context, a fixation cross was presented for a random interval between 1000 and 3000 ms, followed by a face stimulus for 500 ms (see Fig 2). Participants were instructed to press a button to only one type of facial expression per block. Half the participants (randomly assigned) first responded to fearful faces (targets), presented randomly on 70% of trials (30% of the stimuli (nontargets) were happy or neutral faces in alternating blocks, counterbalanced). After eight blocks of 60 trials each, these participants responded to eight blocks of alternating happy targets/fearful nontargets and neutral targets/fearful nontargets. The other half of the participants received the same conditions in reverse order. Trial blocks were separated by short breaks. Prior to data collection, participants completed 20 practice trials per task condition (target classification). Only RTs for correct (Go trials; target present) trials were included in analyses, and criteria for RTs reflecting anticipatory or delayed responding (< 200 ms or >1500 ms, respectively) was set, but no trials were excluded on this basis.

To examine emotion recognition and discrimination, d-prime was calculated by subtracting the z-transformed FA rate from the z-transformed hit rate; d-prime provides a measure of response accuracy that accounts for response bias (e.g., [10]). To assess cognitive control in emotion-laden contexts that may support implicit-controlled emotion regulation, three measures were derived. First, the FA rate was calculated based on incorrect responses, or errors of commission, on No-Go trials. Second, RT on correct, Go trials was calculated to determine the efficiency with which cognitive control was exercised. Finally, in addition to examining RT and FA rate independently, an inverse efficiency score (IES; [31]), a single variable that combines RT and FA rate, was computed. The IES (RT/(1 –FA rate)) accounts for speed/accuracy tradeoffs to index efficient cognitive performance expressed in terms of response time (in ms); higher scores indicate less efficiency [31].

**Electrocortical recording, artifact rejection, and component specification.** Conflict monitoring was assessed through electrocortical recordings and specifically the N200 and P300 ERP components. Electrocortical signals were acquired using a Neuroscan (El Paso, TX, USA) NuAmps Express 40 channel system. Scalp electrode positions were based on the 10–20 international system with a forehead ground and two monopolar mastoid references. The timing, presentation, and synchronization of stimulus presentation and the continuous EEG recording were controlled by Stim2 software (Neuroscan; El Paso, TX). EEGLAB [47] and ERPLAB toolboxes [48] for MATLAB (Mathworks, Natick, MA, USA) were used to process the raw EEG signal. Primarily following guidelines from Luck & Kappenman [49], data were downsampled from 1000Hz to 250Hz and high-pass filtered at .01 Hz to remove linear trends. The data were then submitted to the Cleanline algorithm [50] to statistically prune 60-hz line noise (e.g. from unshielded power outlets) from the raw signal and then the Artifact Subspace Reconstruction algorithm (ASR: [50]) to remove non-stereotypical artifact (e.g. muscle activity, movement). Continuous EEG were locked to onset of stimuli, and data epochs were extracted using a -200 ms to 1500 ms window. The average amplitude between -200 and 0 ms was subtracted from the signal for baseline correction. Data was low-pass filtered at 30Hz and submitted to EEGLab artifact detection algorithms to remove epochs containing extreme values, abnormal trends, improbable data, abnormally distributed data, and abnormal spectra. Average ERP waveforms for each stimulus type were visually inspected in ERPLab. Finally, data was exported in ASCII format for statistical analyses.

According to visual inspection of grand average waveforms, the N200 component was defined as the peak amplitude in a window from 200 to 350 ms post-stimulus-onset at the fronto-central FCz electrode site [32,33,51,52]. The N200 was computed for both Go and No-Go trials to determine whether the component behaved consistently with prior research (i.e., larger for No-Go trials; [33,53]). The P300 was indexed by the peak amplitude at FCz [32,54,55] from 350 to 600 ms from stimulus onset, according to visual inspection of grand average waveforms. The P300 was computed separately for correct Go and No-Go trials.

#### Procedure

An initial online screening assessed study eligibility. Participants attended an initial lab visit to complete written informed consent and several psychological trait measures (not discussed here). At a second lab session, electrocortical and behavioral measures during the emotional go/no-go were collected. After the emotional go/no-go, another task performance measure and passive image viewing task were administered, but are not reported here. After their baseline lab session, participants were then randomly assigned, according to week of the study, to one intervention (MT or BLC) and then completed the CEQ credibility and benefit expectancy measure. Within a week after their third training session, participants returned to the lab to complete a fourth training session and immediately afterwards, the post-test emotional go/no-go, wherein electrocortical and behavioral measures were again collected. Though not reported here, event-based experience sampling data was also collected within a window 6 days prior to and 6 days after the intervention. Supplementary materials and data for this study are available here: https://osf.io/5hngu/?view_only=1ab90d354536433d99cafbb1d383d2c7

#### Statistical analyses

ANOVA models tested intervention differences for the questionnaire measures of credibility and expected benefits, as well as trait and state mindfulness. Multilevel models using Restricted Maximum Likelihood Estimation (REML; [56]) were specified to examine effects of MT vs. BLC on emotional go/no-go behavioral performance and N200 and P300 ERP amplitudes. Each outcome variable was first assessed for normality. In these analyses, we controlled for baseline values when testing effects of MT vs. BLC on each corresponding outcome variable.

## Results

### Preliminary analyses

Preliminary analyses tested for any between-condition differences in demographic variables of age, sex, and race/ethnicity, as well as state and dispositional mindfulness. Separate one-way ANOVAS confirmed no pre-existing differences between MT and BLC on each of these key variables (all *p*s > .10).

### Intervention credibility and expectancy

Scores on the CEQ were first examined to test whether the two conditions differed post-randomization. Due to administrative error, 52 participants had complete data on the CEQ. Two one-way ANOVAs revealed that MT participants scored significantly higher than BLC post-randomization on both the credibility subscale [F(1, 51) = 13.42, *p* < .001] and the expectancy subscale [F(1, 51) = 8.91, *p* = .004]. The CEQ subscales were highly correlated, *r*(1, 51) = .84, *p* < .001. Thus, CEQ subscales were combined and included as a covariate in all models testing intervention effects to account for influences that CEQ scores may have had on intervention outcomes.

### State mindfulness manipulation check

A between-condition difference at posttest on the PQ-M attention subscale was examined in an ANOVA model. PQ-M scores were significantly higher for MT (*M* = 71.13, *SE =* 4.35) than for BLC (*M* = 58.18, *SE =* 4.73) at posttest [F(1, 57) = 4.05, *p* = .048]. Results were highly similar when CEQ scores were included as a covariate, and CEQ credibility and expectancy did not predict PQ-M mindfulness (*p* > .50). Thus, MT appeared to have its desired effect on mindful, focused attention as assessed in the final training session.

### Preliminary analyses of behavioral and neural responses

To provide support for the validity of the emotional go/no-go responses in this study, an initial set of analyses were conducted on baseline emotional go/no-go behavioral and ERP responses for comparison with findings from previous studies using the emotional go/no-go task. Based on such prior research, we expected that emotion recognition and discrimination, indexed by d-prime, would be highest for happy Go stimuli, since happy faces should be easier to discern in the context of fearful No-Go stimuli, and results confirmed this expectation (*p* = .006). Our expectation that d-prime would be lowest for fearful Go stimuli, given that emotional go/no-go posed an additional challenge to discriminating these stimuli (i.e., alternating blocks of neutral and happy faces), was also supported (*p*s < .003).

To test whether our measures of cognitive control in the presence of emotional information were consistent with prior behavioral results (e.g., Hare et al., 2005; Megias et al., 2017), we first examined FA rate, expecting that accuracy for No-Go trials would be lower than for Go trials; Go trials predominate in the emotional go/no-go task, and this increases the need for inhibitory control and thus the possibility of FAs on No-Go trials. Our emotional go/no-go results supported this expectation, with more errors of commission on No-Go trials (3.75% FA rate) than omission on Go trials (0.9% nonresponse rate; *p* < .05).

Regarding approach- and avoidance-related tendencies elicited by facial expressions, we expected that RT to target fearful faces would be slower, due to the need to override the prepotent tendency to avoid unpleasant stimuli. Likewise, we expected that FA rate would be higher to nontarget happy faces, consistent with greater difficulty inhibiting approach responses to pleasant stimuli [16]. Results based on multilevel models revealed a pattern largely consistent with prior research, with RT significantly slower for fearful faces (*M* = 481.01 ms, *SE* = 12.38) compared with happy faces (*M* = 451.58 ms, *SE* = 11.81; *p* = .001), though not significantly slower than for neutral faces (*M* = 468.26 ms, *SE* = 11.73; *p* = .081). As expected, FA rate was significantly higher for happy faces (*M* = .137, *SE* = .009) than neutral (*M* = .095, *SE* = .007) and fearful faces (*M* = .113, *SE* = .009, *p*s < .01). These baseline results revealed approach- and avoidance-related tendencies consistent with other research using the emotional go/no-go task (e.g., [16]).

Testing the validity of the neural indicators of conflict monitoring, a multilevel model using baseline N200 amplitudes revealed significant main effects at electrode site FCz according to both facial expression type [F(1, 56) = 4.36, *p* = .041] and trial type (Go or No-Go) [F(2, 112) = 4.59, *p* = .012]; importantly there was also a significant interaction between face type and trial type [F(2, 112) = 5.52, *p* = .005]. Tukey-Kramer post-hoc tests revealed that the amplitude of the N200 was significantly more negative on No-Go (*M* = -3.60, *SE* = .17) versus Go trials (*M* = -3.46, *SE* = .16). Reflecting the significance of the No-Go N200 as an index of conflict monitoring, N200 amplitudes were larger (more negative) for happy and neutral, compared with fearful No-Go trials (*p*s < .05). Likewise, larger (more negative) Go N200 amplitudes were found for happy, compared with both fearful and neutral faces (*p*s < .05) (cf. [9]). A second multilevel model, on baseline P300 at site FCz, showed that amplitudes were larger for No-Go than for Go trials [F(1, 56) = 19.24, *p* < .001] (cf. [9]). However, there were no differences in No-Go P300 (or Go P300) across facial expressions (*p*s > .60), indicating no emotional moderation of No-Go P300.

### Intervention effects on behavioral and neural responses

In assessing effects of MT versus BLC on emotional go/no-go responses, CEQ credibility and expectancy scores were also tested as a covariate in behavioral and ERP models to be presented here, but they did not predict any pre-post intervention component changes (*ps* > .35), so will not be further discussed.

#### Facial expression recognition and discrimination

Addressing our first prediction concerning MT effects on emotion recognition and discrimination, multilevel models assessed effects of MT relative to BLC on d-prime across facial expressions on Go trials. Consistent with our expectation, MT produced stronger improvement in discrimination across all three facial expressions ([F(1, 58) = 5.16, *p* = .027], *R*^2^ *=* .082; CI = [.14, 2.29]), with no moderation by facial expression type. Specifically, there was no condition difference at baseline, whereas posttest d-prime was higher for MT (*M* = 3.64, *SE* = .08) than for BLC (*M* = 3.47, *SE* = .09). There was also a nonsignificant interaction between intervention condition and baseline d-prime ([F(1, 118) = 3.79, *p* = .053], *R*^2^ *=* .031; CI = [-.62, .01]), suggesting that MT, relative to BLC, may produce marginally greater increases in accuracy among those with lower baseline accuracy (d-prime) scores. Thus, in support of our first hypothesis, MT produced better overall recognition and discrimination of facial expressions, and possibly more so for those with lower manifest ability at baseline.

#### Cognitive control

Addressing our second prediction concerning MT effects on cognitive control efficiency in a socioemotional context, multilevel models first assessed effects of MT relative to BLC on RT and FA separately. The pattern of results indicated that MT produced stronger improvement in both speed and accuracy across all three facial expressions (with no moderation by facial expression type). Specifically, no main effect of intervention was found for FA rate (*p* = .172), but there was an interaction between intervention condition and baseline FA rate, revealing that MT participants had greater accuracy (lower FA rate) at posttest than BLC, particularly for those with higher FA rate at baseline ([F(1, 115) = 4.37, *p* = .039], *R*^2^ *=* .036; CI = [-.42, -.01]). Regarding response speed, a main effect of intervention condition revealed that MT participants had faster RT than BLC, controlling for baseline scores ([F(1, 58) = 5.04, *p* = .028], *R*^2^ *=* .086; CI = [12.99, 226.49]); there was also a significant intervention condition by baseline interaction ([F(1, 115) = 5.49, *p* = .021], *R*^2^ *=* .045; CI = [-.49, -.04]), indicating that MT, relative to BLC, produced faster posttest RT particularly among those with slower baseline RT.

**Fig 3:**
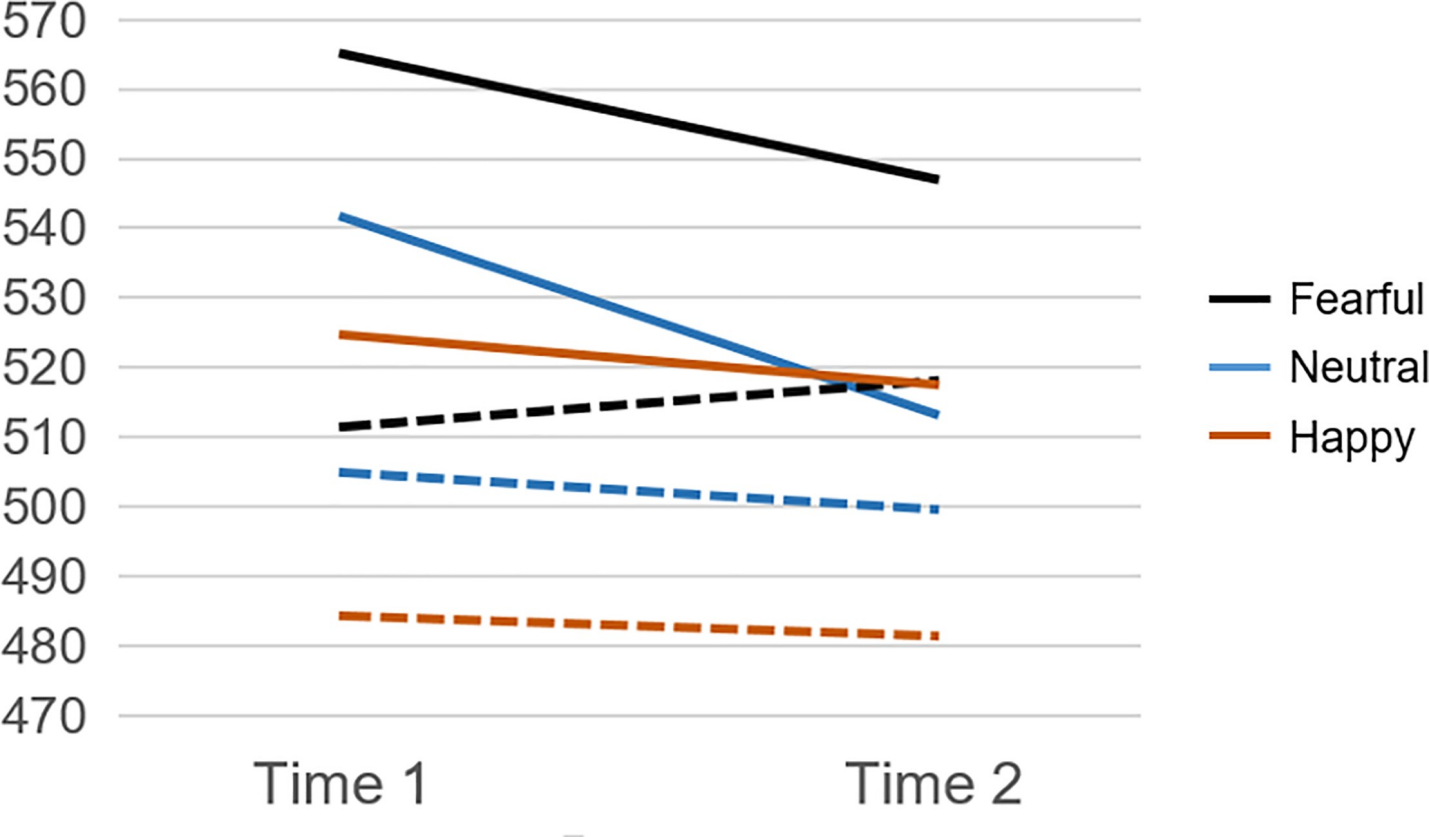
Average inverse efficiency scores (IES) at pretest (Time 1) and posttest (Time 2) for each facial expression as the Go stimulus. Compared with BLC (dashed lines), MT (solid lines) produced a greater decrease in IES overall, indexing improved efficiency of cognitive control on the emotional go/no-go task across neutral, fearful, and happy faces. This effect was most pronounced among those with higher IES at pretest.

To further test the hypothesis that MT would improve cognitive control in a lab-based socioemotional context, inverse efficiency scores (IES), which combine RT and FA into a single variable [31], were examined. As illustrated in Fig 3, a main effect showed that intervention condition significantly predicted IES at posttest after controlling for pretest IES scores ([F(1, 58) = 5.64, *p* = .021], *R*^2^ *=* .088; CI = [24.57, 291.51]). Specifically, IES was lower (reflecting higher cognitive control efficiency) for MT (*M* = 514.19, *SE* = 8.23) than for BLC (*M* = 521.02, *SE* = 9.47); there was no significant interaction with facial expression type. There was also a significant interaction between intervention condition and pretest IES ([F(1, 118) = 6.19, *p* = .016], *R*^2^ *=* .049; CI = [-.56, -.06]), such that MT produced more efficient posttest cognitive control when pretest IES score was higher (less efficient). Thus, in support of our second hypothesis, MT produced better and more efficient cognitive control performance across all three types of facial expressions of emotion, and this effect was most pronounced among MT participants with poorer demonstrated cognitive control at pretest.

#### Conflict monitoring

**Fig 4:**
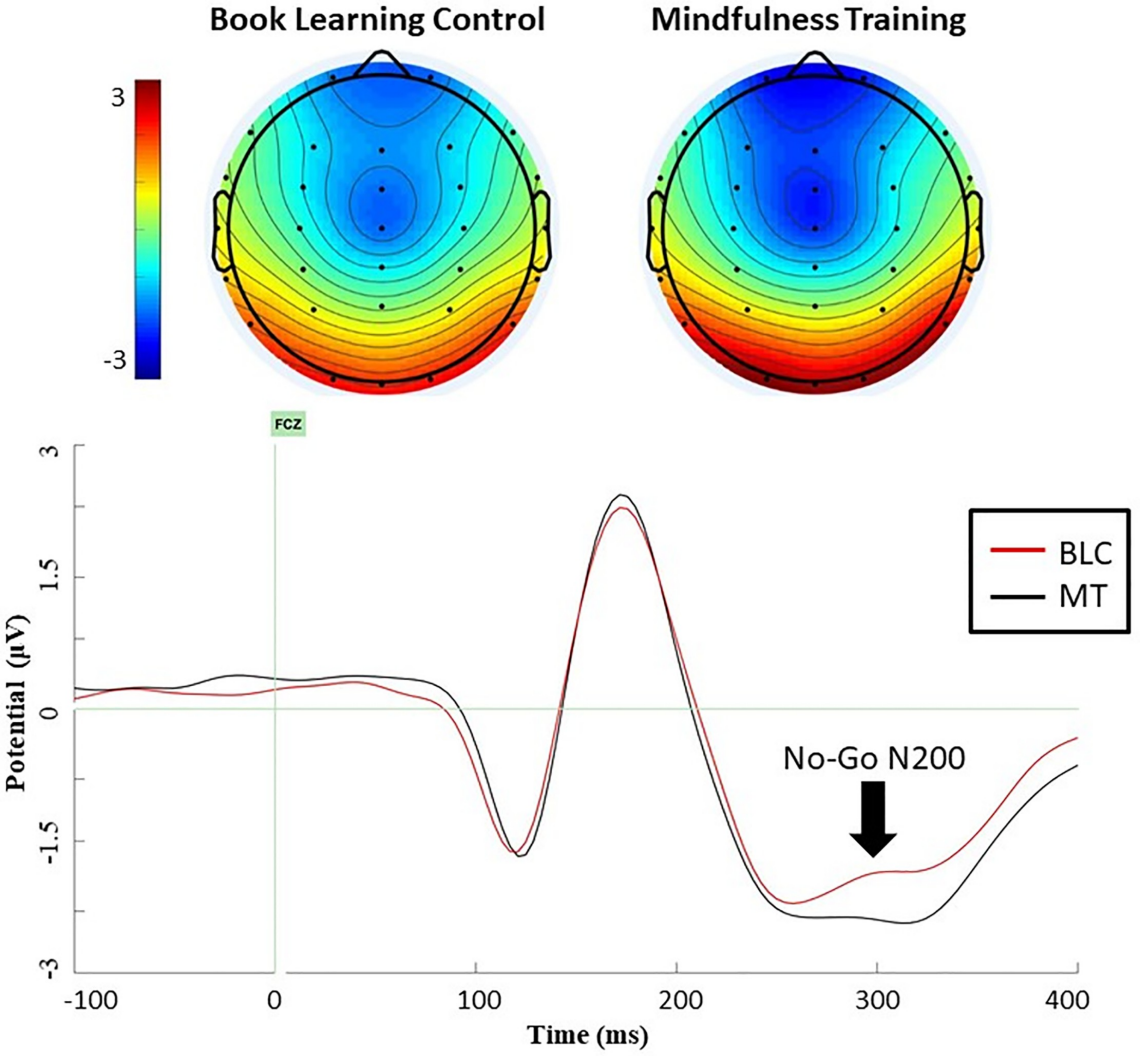
**Top: Scalp topographies at posttest for BLC (left) and MT (right) for 300–350 ms following stimulus onset on No-Go trials during the emotional go/no-go task.** Darker blue indicates more negative (greater) activation. Bottom: Grand average waveform at FCz for BLC (red) and MT (black) from -100 and 400 ms following all stimulus conditions on No-Go trials at posttest, with baseline correction from -200 to 0 ms.

We next tested our third prediction that MT (vs BLC) would produce better monitoring of behavioral goal–prepotent response conflicts, as indexed by ERP outcomes. We expected that relative to BLC participants, MT participants would show a greater pre-post increase in N200 amplitude generally or in No-Go N200 specifically. A multilevel model tested intervention condition as a predictor of N200 at FCz, retaining face type, trial type (Go, No-Go), and pretest N200 amplitude as covariates. Results did not reveal a main effect of intervention condition on posttest N200 across trial type [F(1, 56) = .14, *p* = .711], but there was a significant interaction between intervention condition and trial type ([F(1, 56) = 5.88, *p* = .018], *R*^2^ *=* .095; CI = [-1.43, -.14]), revealing that MT produced larger increases in No-Go N200 specifically and not Go N200. As illustrated in topographic maps and grand average waveforms for the No-Go N200 in Fig 4, participants in MT had more negative No-Go N200 amplitude at posttest (*M* = -3.44, *SE* = .19) relative to those in BLC (*M* = -3.27, *SE* = .21). Further, there was a significant three-way interaction between intervention condition, baseline amplitude, and trial type ([F(1, 282) = 7.97, *p* = .005], *R*^2^ *=* .027; CI = [-.49, -.08]), revealing that the MT effect on No-Go N200 was stronger when this amplitude were smaller at baseline. There was no interaction between intervention condition and face type (*p* = .690). These results provide support for the hypothesis that MT would enhance conflict monitoring as indexed by larger No-Go N200 amplitude, and particularly for those with smaller baseline No-Go N200 amplitudes.

We then tested the effect of MT (vs BLC) on P300 at site FCz in a multilevel model, retaining trial type and pretest P300 amplitude as covariates. Amplitudes at posttest were higher for MT (*M* = 1.14, *SE* = .09) than for BLC (*M* = .98, *SE* = .11), but this difference was not significant [F(1, 56) = .00, *p* = .949], nor was there a significant interaction between intervention condition and trial type [F(1, 56) = .04, *p* = .833]. Thus, and consistent with the preliminary analyses reported earlier, these results did not support the exploratory hypothesis that MT would result in larger No-Go P300.

## Discussion

Mindfulness has long been thought to benefit social interaction (e.g., [57]), but to date little research has targeted the cognitive and neural mechanisms that may underlie social benefits of mindfulness. This experiment showed that relative to a structurally equivalent control condition, a brief 4-session training in a focused attention form of mindfulness meditation produced changes in task-based behavioral and neural measures indicating improvements in dynamically deployed cognitive control in processing and responding to facial expressions of emotion. These findings are among the first to examine effects of MT in the context of socioemotional stimuli, and the first to demonstrate that brief MT can alter cognitive control in ways that may enhance emotion regulation in socioemotional contexts.

Our study relied on behavioral and neural indicators of cognitive control during an emotional go/no-go task to provide an environment wherein effects of MT on three challenges to cognitive control in social situations could be assessed–namely (a) effective identification and discrimination of facial expressions (in this study, happy, neutral, or fearful expressions); (b) adherence to rules for selecting or inhibiting behavior in the face of others’ emotions; and (c) successful monitoring of behavioral goal–prepotent tendency conflicts, whether from task demands or the presence of emotional information. Findings across the behavioral and neural measures supported our expectations that MT would produce benefits relevant to each of these challenges.

Addressing the first challenge, task-based behavioral results supported hypotheses that MT would improve facial expression discrimination, as indicated by higher d-prime scores. This effect of MT was most pronounced for those with lower discrimination (d-prime) scores at baseline, suggesting that training in mindful attention may be more beneficial for those with greater opportunity for change. Task-based behavioral results also supported our prediction concerning the second challenge, that MT would improve the speed and accuracy of behavioral performance on the emotional go/no-go task, including processes of maintaining a task-relevant goal in mind, selecting goal-relevant information, inhibiting prepotent tendencies, and ongoing performance monitoring. Specifically, an interaction between intervention condition and baseline FA rate revealed that MT (vs. BLC) lowered FA rate, and particularly for those with more FAs at baseline. MT also produced faster RT at posttest than BLC. Lastly, when speed and accuracy were combined into a single index of performance efficiency, MT predicted greater improvements than BLC in overall efficiency, and more strongly for those with low efficiency scores at baseline.

Our predictions about the third challenge, concerning conflict monitoring, relied on ERPs, and specifically the N200 component. As with prior go/no-Go research investigating the N200, peak amplitudes for the N200 in this study were higher on No-Go than Go trials. Our results indicated that MT led to greater changes in No-Go N200 amplitude than did BLC. This difference in amplitudes supports the investigation of MT effects on the N200 as a neural index of conflict monitoring, which involves ongoing attention to discrepancies between internally-generated intended behavior and external task demands [32,33].

The effect of MT was limited to the No-Go N200, and the effect was stronger for those with smaller No-Go N200 at baseline. That MT influenced No-Go, but not Go, N200 is therefore consistent with hypothesized effects for MT on conflict monitoring. Better conflict monitoring may help to balance goal pursuit and context sensitivity in dynamic socioemotional environments. The interaction of training with pretest No-Go N200 suggests that among those with more to gain from mindful attention training, brief MT benefited executive attentional capacities to track discrepancies between goal-driven intentions and socioemotional contextual cues. We did not find supportive evidence for our exploratory hypothesis that MT would affect No-Go P300 amplitude (see also [9]), an executive process distinct from No-Go N200. Combined, these ERP findings are consistent with our reasoning that MT-related changes in conflict monitoring (N200) reflect more efficient resolution of conflict that may lessen, or at least leave unchanged, cognitive demands for subsequent behavioral inhibition, indexed by P300.

Taken as a whole, these behavioral and neural results are consistent with the view that MT can foster efficient, context-sensitive cognitive control when responding to facial expressions of pleasant and unpleasant emotions. The findings are generally consistent with those of Quaglia et al. [20], who reported that basic trait mindfulness predicted more time-efficient and accurate cognitive control performance in the emotional go/no go task environment. The more mindful individuals showed amplified N200 and No-Go P300 in that study. However, three neural conflict monitoring results of the present study differed somewhat from those prior results; here, only No-Go N200 was altered by MT, and No-Go P300 was not affected by MT. This might be explained by the possibility that the cognitive control capacities of people higher in basic trait mindfulness differ from those of people receiving a brief (4-day) dose of MT.

The present findings are among the first to demonstrate effects of MT on attention in a social context specifically, an important step toward extending research on mindfulness beyond its chief focus on intrapersonal benefits. The results suggest that mindful emotion regulation could be comparatively less effortful (cf. [21]), since MT modulated neural processes less than 350 ms after the onset of facial expressions of emotion, and may support immediate task goals in a context-sensitive manner, promoting efficient deployment of top-down attention when needed for effective regulation. The findings also advance our understanding of neural and cognitive mechanisms of MT more generally, and support the idea that efficient cognitive control may be key to understanding beneficial effects of MT on cognition and behavior (cf. [13,14]). Given that social situations place unique demands on attention for which mindfulness appears well-suited, research can build on these findings to better understand socioemotional benefits of MT.

### Limitations and future directions

It is important to acknowledge that assessing effects of brief MT in this study did not allow for a disentangling of trait-like and state-like effects of MT, as the post-training emotional go/no-go outcomes were assessed once, immediately following the 4^th^ training session. Future research examining MT effects on cognitive control may assess whether effects are evident when follow-up assessment(s) take place days or weeks following training. A second limitation of the study is that significant differences in expected benefits and credibility were found between intervention conditions post-randomization, with greater expected benefits and more credibility reported by those in the MT condition. A number of critical nonspecific intervention effects were controlled via BLC (instructor time, session and intervention length, etc.), but this control condition could not account for placebo effects due to beliefs about learning mindfulness meditation. It is possible that BLC participants were less motivated than MT participants to perform well on the EGNG task at posttest. All analyses testing intervention effects therefore controlled for credibility and expectancy, and no differences in outcomes were found. Nonetheless, future research should use more closely matched active control conditions to more directly address credibility and expected benefit (e.g., sham-mindfulness meditation; [28,29]).

A third limitation of the study was that the final sample size for analyses was lower than optimal, per *a priori* power analysis. To indicate the range of likely values in the population, we reported 95% confidence intervals for each primary finding. The sample was also quite homogenous in terms of sex, race/ethnicity, and relationship status, potentially limiting the generalizability of the results. Regarding the fact that all participants were in romantic relationships, such people may be more skilled in cognitive control in intimate socioemotional contexts, which may benefit their social responsivity more generally. A valuable test of the generalizability of the present results would be to determine whether they replicate in a larger and more diverse sample among those not coupled and, of clinical relevance, among those who have had cognitive control difficulties in romantic and other relationships.

Further research is also called for to examine whether the effects on cognitive control in socioemotional contexts observed here are specific to focused attention-based MT or also extend to an open monitoring style of MT. The latter is characterized by a receptive, non-judgmental stance on momentary experience that bears similarities to exposure [58]; this form of training may benefit responding to challenging emotions in social contexts. Research could examine the potential of longer-term MT to exert impact on cognitive control in socioemotional contexts, which may moreover help to disentangle state versus trait effects of MT. It will also be important to determine whether the enhanced cognitive control among mindfulness trainees observed here extends beyond laboratory task contexts. Our reliance on the emotional go/no-go task allowed for investigation of MT’s effects on attention in a relatively fixed socioemotional context–that is, using static facial expressions–compared with the dynamic nature of real-world social situations wherein there may be (and often are) interaction effects between emotional actors and perceivers that influence cognitive control and social behavior. Future research could extend this study by examining whether cognitive control helps to explain benefits of mindfulness in dynamic socioemotional contexts, such as during live social interactions (e.g., [59]). Finally, future research could examine whether the cognitive control effects found here are specific to socioemotional contexts or reflect broader cognitive control capacities.

## Concluding remarks

The ubiquity of emotional experience and expression across varied social contexts leaves little doubt that people’s social and emotional lives are strongly interdependent, hinging on others’ (and one’s own) ongoing responses. This study of MT and its effects on neural and behavioral responses in socioemotional contexts suggests that attention deployment is integral to effective cognitive control of behavior in social interactions. While Buddhist psychological theory has long emphasized the relevance of mindful attention to social life [57], research on mindfulness has focused primarily on intrapersonal processes outside of social contexts. The present findings are the first to demonstrate effects of brief MT on cognitive control in the face of others’ emotions, and strengthen the perspective that social benefits of mindfulness may be understood in part through early top-down attention deployment. This efficient use of attention could help to drive behavior regulation in dynamic socioemotional contexts that is timely and context-sensitive. Future research can build on these findings to better understand how MT may promote emotional and social well-being by explicitly examining underlying mechanisms of cognitive control.

## References

[1] Duncan J. An adaptive coding model of neural function in prefrontal cortex. Nat Rev Neurosci. 2001;2(11):820–9. 10.1038/35097575 11715058

[2] Haas D, Keel C. An integrative theory of prefrontal cortex function. Annu Rev Neurosci. 2003;41(1):117–53. 10.1146/annurev.neuro.24.1.167 11283309

[3] Hofmann W, Schmeichel BJ, Baddeley AD. Executive functions and self-regulation. Trends Cogn Sci. 2012;16(3):174–80. 10.1016/j.tics.2012.01.006 22336729

[4] Robinson MD, Schmeichel BJ, Inzlicht M. A cognitive control perspective of self-control strength and its depletion. Soc Personal Psychol Compass. 2010;4(3):189–200.

[5] Slagter HA, Davidson RJ, Lutz A. Mental training as a tool in the neuroscientific study of brain and cognitive plasticity. Front Hum Neurosci. 2011;5:17 10.3389/fnhum.2011.00017 21347275 PMC3039118

[6] Pessoa L. On the relationship between emotion and cognition. Nat Rev Neurosci. 2008;9(2):148–58. 10.1038/nrn2317 18209732

[7] Pessoa L. How do emotion and motivation direct executive control? Trends Cogn Sci. 2009;13(4):160–6. 10.1016/j.tics.2009.01.006 19285913 PMC2773442

[8] Perales JC. Corrigendum: Emotional and non-emotional pathways to impulsive behavior and addiction. Front Hum Neurosci. 2014;8:411. 10.3389/fnhum.2013.00043 PMC3578351 23441001

[9] Megías A, Gutiérrez-Cobo MJ, Gómez-Leal R, Cabello R, Fernández-Berrocal P. Performance on emotional tasks engaging cognitive control depends on emotional intelligence abilities: An ERP study. Sci Rep. 2017;7(1):16446 10.1038/s41598-017-16657-y 29180769 PMC5703978

[10] Tottenham N, Hare TA, Casey BJ. Behavioral assessment of emotion discrimination, emotion regulation, and cognitive control in childhood, adolescence, and adulthood. Front Psychol. 2011;2:39 10.3389/fpsyg.2011.00039 21716604 PMC3110936

[11] Anālayo B. Satipaṭṭhāna: the direct path to realization. Birmingham, England: Windhorse; 2003.

[12] Todd RM, Cunningham WA, Anderson AK, Thompson E. Affect-biased attention as emotion regulation. Trends Cogn Sci. 2012;16(7):365–72. 10.1016/j.tics.2012.06.003 22717469

[13] Tang YY, Hölzel BK, Posner MI. The neuroscience of mindfulness meditation. Nat Rev Neurosci. 2015;16(4):213–25. 10.1038/nrn3916 25783612

[14] Teper R, Segal Z V., Inzlicht M. Inside the mindful mind: how mindfulness enhances emotion regulation through improvements in executive control. Curr Dir Psychol Sci. 2013;22(6):449–54.

[15] Roelofs K, Minelli A, Mars RB, Van Peer J, Toni I. On the neural control of social emotional behavior. Soc Cogn Affect Neurosci. 2008;4(1):50–8. 10.1093/scan/nsn036 19047074 PMC2656885

[16] Hare TA, Tottenham N, Davidson MC, Glover GH, Casey BJ. Contributions of amygdala and striatal activity in emotion regulation. Biol Psychiatry. 2005;57(6):624–32. 10.1016/j.biopsych.2004.12.038 15780849

[17] Ekman P. Emotions revealed: recognizing faces and feelings to improve communication and emotional life. 2^nd^ ed New York: Henry Holt; 2007.

[18] Braunstein LM, Gross JJ, Ochsner KN. Explicit and implicit emotion regulation: A multi-level framework. Soc Cogn Affect Neurosci. 2017;12(10):1545–57. 10.1093/scan/nsx096 28981910 PMC5647798

[19] Vogel EK, Luck SJ. The visual N1 component as an index of a discrimination process. Psychophysiology. 2000;37(2):190–203. 10731769

[20] Quaglia JT, Goodman RJ, Brown KW. Trait mindfulness predicts efficient top-down attention to and discrimination of facial expressions. J Pers. 2016;84(3):393–404. 10.1111/jopy.12167 25676934

[21] Sheppes G, Gross JJ. Is timing everything? Temporal considerations in emotion regulation. Pers Soc Psychol Rev. 2011;15(4):319–31. 10.1177/1088868310395778 21233326

[22] Jha AP, Krompinger J, Baime MJ. Mindfulness training modifies subsystems of attention. Cogn Affect Behav Neurosci. 2007;7(2):109–19. 17672382 10.3758/cabn.7.2.109

[23] Fan J, McCandliss BD, Sommer T, Raz A, Posner MI. Testing the efficiency and independence of attentional networks. J Cogn Neurosci. 2002;14(3):340–7. 10.1162/089892902317361886 11970796

[24] Tang Y-Y, Ma Y, Wang J, Fan Y, Feng S, Lu Q, et al Short-term meditation training improves attention and self-regulation. Proc Natl Acad Sci. 2007;104(43):17152–6. 10.1073/pnas.0707678104 17940025 PMC2040428

[25] Teper R, Inzlicht M. Meditation, mindfulness and executive control: The importance of emotional acceptance and brain-based performance monitoring. Soc Cogn Affect Neurosci. 2012;8(1):85–92. 10.1093/scan/nss045 22507824 PMC3541488

[26] Lutz A, Slagter HA, Dunne JD, Davidson RJ. Attention regulation and monitoring in meditation. Trends Cogn Sci. 2008;12(4):163–9. 10.1016/j.tics.2008.01.005 18329323 PMC2693206

[27] Allen M, Dietz M, Blair KS, van Beek M, Rees G, Vestergaard-Poulsen P, et al Cognitive-affective neural plasticity following active-controlled mindfulness intervention. J Neurosci. 2012;32(44):15601–10. 10.1523/JNEUROSCI.2957-12.2012 23115195 PMC4569704

[28] Zeidan F, Johnson SK, Gordon NS, Goolkasian P. Effects of brief and sham mindfulness meditation on mood and cardiovascular variables. J Altern Complement Med. 2010;16(8):867–73. 10.1089/acm.2009.0321 20666590

[29] Zeidan F, Emerson NM, Farris SR, Ray JN, Jung Y, McHaffie JG, et al Mindfulness meditation-based pain relief employs different neural mechanisms than placebo and sham mindfulness meditation-induced analgesia. J Neurosci. 2015;35(46):15307–25. 10.1523/JNEUROSCI.2542-15.2015 26586819 PMC4649004

[30] Zeidan F, Adler-Neal AL, Wells RE, Stagnaro E, May LM, Eisenach JC, et al Mindfulness-meditation-based pain relief is not mediated by endogenous opioids. J Neurosci. 2016;36(11):3391–7. 10.1523/JNEUROSCI.4328-15.2016 26985045 PMC4792946

[31] Bruyer R, Brysbaert M. Combining speed and accuracy in cognitive psychology: Is the inverse efficiency score (IES) a better dependent variable than the mean reaction time (RT) and the percentage of errors (PE)?. Psychol Belg. 2011;51(1):5–13.

[32] Donkers FCL, Van Boxtel GJM. The N2 in go/no-go tasks reflects conflict monitoring not response inhibition. Brain Cogn. 2004;56(2):165–76. 10.1016/j.bandc.2004.04.005 15518933

[33] Nieuwenhuis S, Yeung N, Van Den Wildenberg W, Ridderinkhof KR. Electrophysiological correlates of anterior cingulate function in a go/no-go task: effects of response conflict and trial type frequency. Cogn Affect Behav Neurosci. 2003;3(1):17–26. 12822595 10.3758/cabn.3.1.17

[34] Huster RJ, Westerhausen R, Pantev C, Konrad C. The role of the cingulate cortex as neural generator of the N200 and P300 in a tactile response inhibition task. Hum Brain Mapp. 2010;31(8):1260–71. 10.1002/hbm.20933 20063362 PMC6871040

[35] Polich J. Updating P300: An integrative theory of P3a and P3b. Clin Neurophysiol. 2007;118(10):2128–48. 10.1016/j.clinph.2007.04.019 17573239 PMC2715154

[36] Quaglia JT, Goodman RJ, Brown KW. From mindful attention to social connection: the key role of emotion regulation. Cogn Emot. 2015;29(8):1466–74. 10.1080/02699931.2014.988124 25496330

[37] Cohen J. A power primer. Psychol Bull. 1992;112(11):155. 19565683 10.1037//0033-2909.112.1.155

[38] Zeidan F, Johnson SK, Diamond BJ, David Z, Goolkasian P. Mindfulness meditation improves cognition: evidence of brief mental training. Conscious Cogn. 2010;19(2):597–605. 10.1016/j.concog.2010.03.014 20363650

[39] Gunstad J, Paul RH, Cohen RA, Tate DF, Spitznagel MB, Gordon E. Elevated body mass index is associated with executive dysfunction in otherwise healthy adults. Compr Psychiatry. 2007;48(1):57–61. 10.1016/j.comppsych.2006.05.001 17145283

[40] Chapman LJ, Chapman JP. The measurement of handedness. Brain Cogn. 1987;6(2):175–83. 3593557 10.1016/0278-2626(87)90118-7

[41] Propper RE, Pierce J, Geisler MW, Christman SD, Bellorado N. Asymmetry in resting alpha activity: Effects of handedness. Open J Med Psychol. 2012;01(4):86–90.

[42] White G. The natural history of Selborne. 1948.

[43] Devilly GJ, Borkovec TD. Psychometric properties of the credibility/expectancy questionnaire. J Behav Ther Exp Psychiatry. 2000;31(2):73–86. 11132119 10.1016/s0005-7916(00)00012-4

[44] Walach H, Buchheld N, Buttenmüller V, Kleinknecht N, Schmidt S. Measuring mindfulness—the Freiburg mindfulness inventory (FMI). Pers Individ Dif. 2006;40(8):1543–55.

[45] Del Re AC, Flückiger C, Goldberg SB, Hoyt WT. Monitoring mindfulness practice quality: An important consideration in mindfulness practice. Psychother Res. 2013;23(1):54–66. 10.1080/10503307.2012.729275 23046287

[46] Tottenham N, Tanaka JW, Leon AC, McCarry T, Nurse M, Hare TA, et al The NimStim set of facial expressions: judgments from untrained research participants. Psychiatry Res. 2009;168(3):242–9. 10.1016/j.psychres.2008.05.006 19564050 PMC3474329

[47] Delorme A, Makeig S. EEGLAB: an open source toolbox for analysis of single-trial EEG dynamics. J Neurosci Methods. 2004;134(1):9–21. 10.1016/j.jneumeth.2003.10.009 15102499

[48] L-C J., S.J. L. ERPLAB: An open-source toolbox for the analysis of event-related potentials. Front Hum Neurosci. 2014;8:213 10.3389/fnhum.2014.00213 24782741 PMC3995046

[49] Luck SJ, Kappenman ES. The Oxford handbook of event-related potential components. New York: Oxford University Press; 2011.

[50] Mullen T, Kothe C, Chi YM, Ojeda A, Kerth T, Makeig S, et al Real-time modeling and 3D visualization of source dynamics and connectivity using wearable EEG. Conf. Proc. IEEE Eng. Med. Biol. Soc. 2013;2184–7. 10.1109/EMBC.2013.6609968 24110155 PMC4119601

[51] Bruin KJ, Wijers AA. Inhibition, response mode, and stimulus probability: a comparative event-related potential study. Clin Neurophysiol. 2002;113(7):1172–82. 12088714 10.1016/s1388-2457(02)00141-4

[52] Zhang W, Lu J. Time course of automatic emotion regulation during a facial Go/Nogo task. Biol Psychol. 2012;89(2):444–9. 10.1016/j.biopsycho.2011.12.011 22200654

[53] Amodio DM, Master SL, Yee CM, Taylor SE. Neurocognitive components of the behavioral inhibition and activation systems: implications for theories of self-regulation. Psychophysiology. 2008;45(1):11–9. 10.1111/j.1469-8986.2007.00609.x 17910730

[54] Hagen GF, Gatherwright JR, Lopez BA, Polich J. P3a from visual stimuli: task difficulty effects. Int J Psychophysiol. 2006;59(1)8–14. 10.1016/j.ijpsycho.2005.08.003 16253363

[55] Katayama J, Polich J. Stimulus context determines P3a and p3b. Psychophysiology. 1998;35(1):23–33. 9499703

[56] Raudenbush SW. In: Edwards L. Applied analysis of variance in behavioral science Hierarchical linear models and experimental design. New York: Marcel Decker; 1993 pp. 137–459.

[57] Williams P. Mahayana Buddhism: The doctrinal foundations. 2nd ed United Kingdom: Routledge; 2008.

[58] Hölzel BK, Lazar SW, Gard T, Schuman-Olivier Z, Vago DR, Ott U. How does mindfulness meditation work? Proposing mechanisms of action from a conceptual and neural perspective. Perspect Psychol Sci. 2011;6(6):537–59. 10.1177/1745691611419671 26168376

[59] Barnes S, Brown KW, Krusemark E, Campbell WK, Rogge RD. The role of mindfulness in romantic relationship satisfaction and responses to relationship stress. J Marital Fam Ther. 2007;33(4):482–500. 10.1111/j.1752-0606.2007.00033.x 17935531

